# The Chicken Egg: An Advanced Material for Tissue Engineering

**DOI:** 10.3390/biom14040439

**Published:** 2024-04-04

**Authors:** Yuli Zhang, Hieu M. Pham, Simon D. Tran

**Affiliations:** 1 Faculty of Dental Medicine and Oral Health Sciences, McGill University, 3640 University Street, Montreal, QC H3A 0C7, Canada; yuli.zhang@mail.mcgill.ca (Y.Z.); hieu_pham@urmc.rochester.edu (H.M.P.); 2 Department of Periodontology, Eastman Institute for Oral Health, University of Rochester Medical Center, 625 Elmwood Avenue, Rochester, NY 14620, USA

**Keywords:** egg, egg white, egg yolk, eggshell, eggshell membrane, biomaterial, hydrogel, tissue engineering

## Abstract

The chicken egg, an excellent natural source of proteins, has been an overlooked native biomaterial with remarkable physicochemical, structural, and biological properties. Recently, with significant advances in biomedical engineering, particularly in the development of 3D in vitro platforms, chicken egg materials have increasingly been investigated as biomaterials due to their distinct advantages such as their low cost, availability, easy handling, gelling ability, bioactivity, and provision of a developmentally stimulating environment for cells. In addition, the chicken egg and its by-products can improve tissue engraftment and stimulate angiogenesis, making it particularly attractive for wound healing and tissue engineering applications. Evidence suggests that the egg white (EW), egg yolk (EY), and eggshell membrane (ESM) are great biomaterial candidates for tissue engineering, as their protein composition resembles mammalian extracellular matrix proteins, ideal for cellular attachment, cellular differentiation, proliferation, and survivability. Moreover, eggshell (ES) is considered an excellent calcium resource for generating hydroxyapatite (HA), making it a promising biomaterial for bone regeneration. This review will provide researchers with a concise yet comprehensive understanding of the chicken egg structure, composition, and associated bioactive molecules in each component and introduce up-to-date tissue engineering applications of chicken eggs as biomaterials.

## 1. Introduction

Tissue engineering and regenerative medicine (TERM) have emerged as promising approaches to address the limitations of conventional treatments for tissue and organ damage, offering new therapeutic techniques that have revolutionized the field of biomedical research [1,2,3]. TERM involves the integration of cells, biomaterials, and bioactive molecules to synthesize a network of biologically functional tissue substitutes or to stimulate the body’s natural regenerative processes [4]. Currently, TERM is a relatively novel field with many avenues left to explore. One avenue of particular interest is appropriate biomaterial scaffold selection [5,6]. Biomaterial scaffolds serve to contain, protect, and transport cells and their necessary bioactive molecules and essential nutrients [7]. The scaffold should ideally mimic the specific native tissue microenvironment for the cell of interest, conducive to their proliferation, differentiation, migration, reorganization, and survivability [8,9]. Thus, the selection of an appropriate biomaterial involves careful consideration of the material’s biological, mechanical, and physiochemical properties, including but not limited to the availability of attachment substrates for cells, diffusion capability, biocompatibility, degradation rate, stiffness, tuneability, and cost [10,11,12,13].

Traditional biomaterials used range from ceramics, synthetic and natural polymers, metals and alloys, or even in varying combinations, all of which will offer distinct advantages and disadvantages depending on the desired characteristic and purpose [14,15]. In recent light, the chicken egg has garnered attention for its viability as a biomaterial for TERM research due to its vast advantages, including accessibility, affordability, biocompatibility, biodegradability, and inherent bioactivity [16,17]. Moreover, there are fewer ethical concerns due to the fact that the commercially available eggs are mostly unfertilized [18,19,20].

The four major layers of the chicken egg are the eggshell (ES), eggshell membrane (ESM), egg white (EW), and egg yolk (EY). These components collectively possess a rich array of proteins, minerals, and growth factors that contribute to unique physiochemical and biological properties [21,22]. Studies have shown that each component has the potential to be used as a biomaterial on its own; however, researchers can also extract, modify, and use each specific component in combination with other biomaterials to enhance bioactivity, mechanical strength, and/or provide controlled release capabilities [23,24].

Despite the promising prospects of chicken-egg-based biomaterials, challenges and limitations exist. For instance, the variability in egg composition from egg-to-egg and batch-to-batch and potential allergenicity need to be carefully addressed. Moreover, the translation of egg-based biomaterials from preclinical studies to clinical applications requires a comprehensive understanding and optimization of their physicochemical properties, biocompatibility, and long-term stability [21].

To our knowledge, there is limited research connecting the basic introduction of the chicken egg and its chemical and biological properties to its application in tissue engineering. This paper aims to provide a comprehensive overview of the use of chicken eggs as a biomaterial for tissue engineering and regenerative medicine research. This paper will discuss the unique structure, composition, and biological properties of each chicken egg component and highlight their current state of research and applications. By describing the egg’s properties from the perspective of tissue engineering, regenerative medicine, and as a biomaterial, providing specific examples of its applications, readers can gain insight into its vast innovative potential.

### 1.1. The Chicken Egg’s Structure and Physicochemical Properties

The chicken egg consists of four main components: the eggshell, eggshell membrane (ESM), egg white (EW), and egg yolk (EY). Each component serves a unique purpose in protecting and supporting the developing embryo if fertilized [21].

The most outer layer is the eggshell, which is a thin, hard, and brittle covering of approximately 0.3 mm in thickness, primarily composed of calcium carbonate (CaCO_3_) and phosphate. The shell serves to provide structural integrity and protect the inner components (yolk, white, and embryo) from mechanical impact, microbial contamination, and dehydration [25]. The ES is composed of three layers: the most outer surface (cuticle), a spongy (calcareous) layer, and an inner lamellar (mamillary layer). The spongy and inner lamellar layer together form a porous matrix of protein fibers and calcite crystal. This porous architecture (7000–17,000 pores per egg) allows for oxygen, carbon dioxide, and moisture to permeate into the egg. The outer cuticle layer is covered with mucin proteins, which effectively plug and seal the pores, physically keeping bacteria out. Furthermore, it has been shown that these proteins also exhibit antimicrobial activity [25,26]. Structurally, the chicken egg forms with two distinct poles: a blunted end and a pointed end [27]. The physiochemical properties of the two inner layers remain relatively consistent across batches of eggs and over time, with some minor variations; however, studies have shown that the quality of the cuticle layer changes depending on the hen’s age at the time of egg formation; decline in the quality of the cuticle layer increases the egg’s susceptibility to microbial invasion and physiochemical changes. [25]. Its rigid matrix and crystalline structures provide it with ideal properties for use in powder and/or microparticle form for bone regeneration [21].

The next component is the ESM, which has several functions. The ESM is a thin fibrous protein network just beneath the ES. During egg development, it acts as the structural foundation for the formation and calcification of the shell. It also acts as an additional physical barrier to microbial invasion, supports the formation of enzymes and proteins, and compartmentalizes the egg white component, preventing it from the mineralizing processes that occur in the eggshell. Like the ES, the ESM is also composed of three distinct substructure layers: the most outer layer (outer shell membrane), the middle layer (inner shell membrane), and the most inner layer (limiting membrane) [18,28,29]. Between the outer shell membrane and inner shell membrane, an air cell can be found on the blunt end of the egg. Because the ES is porous, this is where gas exchange typically occurs. This air cell enlarges as the egg cools after being laid and also enlarges over time as the egg undergoes gas exchange and its water content evaporates. Due to its ability to act as a mesh foundation for ES formation and high protein content, ESM has reportedly been used as a whole membrane and in powdered form for bone, cartilage, skin, and vasculature regeneration studies [21].

Below the ESM is the EW. EW is a clear gel-like component of the chicken egg. Like the ES and ESM, the EW component also serves as a physical barrier against bacteria infiltration. Additionally, several proteins in this component, such as conalbumin and lysozyme, have antibacterial properties [18]. The EW has two clear phases: an inner thick albumin and outer thin albumen. The thick albumen has a much more viscous property and forms a capsule directly around the EY and its membrane. Meanwhile, the thin albumin surrounds the thick albumin, closer to the ESM. The thin albumen has a higher water content and functions to provide moisture to the yolk and develop embryos if fertilized. Two opaque EW spiral filaments that extend from the polar ends of the EY and attach to the ES, called the chalazae, form part of the EW. These filaments suspend and maintain the EY in the center of the egg, providing stability and a protective mechanism against the inertia of external forces. Unlike the ES, the physiochemical properties of EW can vary greatly over time depending on various factors, including atmospheric temperature and storage duration. For example, studies have shown that the pH of EW ranges from 7.6 to 9.7. The pH initially increases rapidly after the egg is laid; it becomes more alkalotic over time, particularly with increased temperatures. However, after even longer incubation periods, the pH of the whites eventually decreases. This correlation is directly related to the diffusion of carbon dioxide out of the egg [30]. Other notable physiochemical properties of EW include its high water-holding capacity, foaming properties, emulsifying properties, and heat-induced gelation properties. These properties are attributed to its diverse protein composition (over 150 different proteins). However, its gel-like properties can be altered through changes in pH and temperature and the presence of salts [31,32]. Due to its unique properties, it is reportedly being used in powder and hydrogel form for TERM research [21].

**Figure 1:**
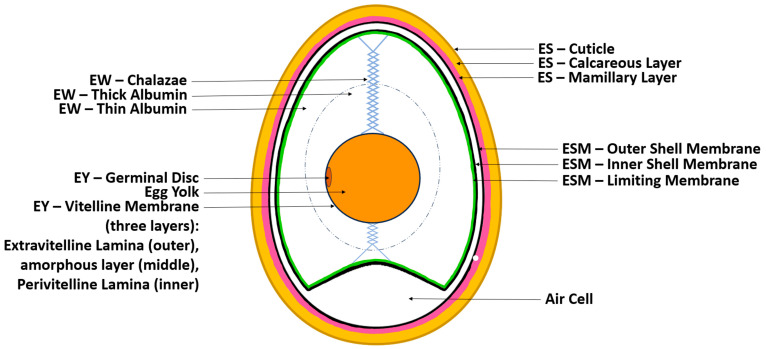
Anatomy of a chicken egg, illustrating its components: egg white (EW), egg yolk (EY), eggshell (ES), and eggshell membrane (ESM).

The most inner layer of the chicken egg is the EY, which is enclosed by a transparent proteinaceous membrane called the vitelline membrane. This membrane is also composed of three sublayers, which include an amorphous middle layer encapsulated between a more fibrous inner (perivitelline lamina) and outer layer (extravitelline lamina) [27,33]. This membrane functions as the final barrier against bacterial invasions and also functions to maintain the shape of the yolk and compartmentalize it from the white. Within the vitelline membrane is the EY itself—a pigmented homogenized fluid body that serves as a source of nutrients for the developing embryo (if fertilized). The EY has two distinct phases: one being a clear yellow aqueous phase (plasma), which contains a majority of the yolk’s lipids and soluble proteins, and the other being an insoluble phase (granules). Due to its high lipid contents, it has excellent gelation properties that can also be modified through temperature. The pH of the EY component is relatively more robust with changes in temperature and over time compared to EW and is reported to have a range of approximately 5.9 to 6.5 [30]. Various components of the EY have been isolated and used in gel or oil form [21] (Figure 1).

### 1.2. The Chicken Egg’s Composition

The content of the chicken egg is capable of nurturing a chicken embryo; therefore, the egg contains all necessary nutrients and ideal composition to support cellular activity and biological growth. This diverse composition ultimately contributes to the egg’s unique structure, physicochemical properties, and biological activities [21,34]. The whole egg is mainly composed of water (74.4%), proteins (12.3%), and lipids (11.6%). It also contains small amounts of carbohydrates (1%), many minerals and trace elements (phosphorus, sodium, strontium, potassium, sulfur, iron, copper, silica, chlorine), essential amino acids, antioxidants, and every vitamin with the exception of vitamin C. However, it is important to note that variations in the composition exist and may vary depending on chicken strain, age, and environmental factors [33,35,36]. The average proportion of total egg weight of each component is as follows: ES (9%), ESM (<0.5%), EW (62%), and EY (29%) [33,36].

The shell is composed of 95% inorganic minerals, 3.5% organic material, and 1.5% water. It has been reported that calcium carbonate makes up to 98.5% of the inorganic minerals. Other inorganic minerals found in the shell include calcium phosphate (Ca_3_(PO_4_)_2_), magnesium carbonate (MgCO_3_), and trace amounts of other ions including sodium, strontium, potassium, sulfur, and silica [18,21,33]. The organic aspect of the ES comes from the outer cuticle layer, which is composed of 90% glycoproteins, but also contains 4% carbohydrates, 3% lipids, and 3% hydroxyapatite crystals [37].

The fibrous network of ESM is composed of 90% proteins, many of which resemble human extracellular matrix (ECM) such as collagen type I, collagen type V, collagen type X, glucosamine, proteoglycan, desmosine, keratin, hyaluronic acid, and sialic acid. The various collagen types represent approximately 10% of the total proteins. They also contain approximately 3% lipids and 2% carbohydrates. Of its composition, there are also bioactive and functional molecules including hyaluronic acid, amines, amides, and carboxylic acids [21].

The EW makes up the majority of the total egg weight at approximately 62% and makes up 56% of the protein found in eggs. In addition to protein, the EW component also contains carbohydrates, vitamins, minerals, and water. The protein component is made up of more than 150 proteins. These proteins can be categorized as main proteins or minor proteins. Ovalbumin is a major protein that makes up 54% of the total EW proteins while other major proteins include ovotransferrin (12%), ovomucoid (11%), globulins (8%), ovomucin (3.5%), and lysozyme (3.5%) [21,33,38]. Some prominent minor proteins include ovoinhibitor (1.5%), ovoglycoprotein (1%), flavoprotein (0.8%), ovomacroglobulin (0.5%), cystatin (0.05%), and avidin (0.05%), which offer key biological activities (further discussed below).

The innermost component of the chicken egg, the EY, has a mean total egg weight of 29%. EY is composed of 50% water, 30% lipids, 16% proteins, 4% carbohydrates, and various micronutrients including vitamins (A, B, D, E, K,) and essential minerals (calcium, iron, zinc, magnesium, potassium, and sodium) [21,33]. The aqueous phase contains 90% of the EY’s total lipids, a majority of it being low-density lipids (LDLs), and approximately 50% of the EY’s total proteins. Conversely, the insoluble phase contains the remaining 10% lipids, mostly as high-density lipids (HDLs), and the other half of the EY’s total proteins. Some notable proteins found in the EY with biological activity include livetin, phosvitin, and immunoglobulins (further discussed below) [21,39].

### 1.3. The Chicken Egg’s Biological Activity

While the eggshell is often overlooked due to its relative lack of nutrients, it does have a place in regenerative medicine. For example, due to the presence of calcium phosphate, ES has been shown to have high bioactivity. It also possesses biodegradable properties, making it suitable for biomaterial research. Furthermore, the trace elements found in the ES have been shown to be capable of promoting angiogenesis and osteogenesis. Evidently, studies have shown its use in bone tissue engineering research as it can serve as a biocompatible scaffold for osteogenesis, and cell attachment and proliferation [21]. In an in vitro study by Dadhich (2016), it was shown that naturally derived calcium phosphate from ES led to greater cellular activity and growth compared to synthetic calcium phosphate, suggesting that the unique composition of ES has great biological activity and potential uses in TERM research [40].

Similar to the other components of the egg, the ESM also provides antibacterial, biodegradability, and biocompatibility properties. In an in vitro study, it has been suggested that the ESM is capable of binding and destroying bacterial cell membranes [41]. Furthermore, due to its high collagen composition, ESM resembles human ECM and thus has the ability to induce cellular adhesion, migration, and proliferation [21]. Though limited to mice studies, other biological activities of the ESM such as wound healing, anti-inflammatory, and anti-obesity have also been observed [41].

EW has a wide variety of functions and biological properties because of its diverse functional protein profile. As with all components of the chicken egg, EW exhibits biocompatibility, biodegradability, and antibacterial properties. Ovalbumin, the most abundant protein in EW, plays a role in cell adhesion and attachment and has antioxidant properties [18]. Conalbumin, ovotransferrin, and lysozyme are three other major proteins of EW that also demonstrate antibacterial properties. It is noteworthy that lysozyme, among the other antibacterial proteins, demonstrates broad-spectrum antibacterial specificity, and can also moderate cells from malignant transformation [18,34]. Another major protein of EW is ovomucoid, which is highly significant as it is the primary source of egg allergies in humans [21]. Like ESM, EW has a resemblance to human ECM and thus exhibits ECM-like properties, which include adsorbing and modulating the diffusion of growth factors and other nutrients, acting as a substrate for cellular attachment, and protecting cells from enzymatic degradation. Numerous in vivo studies further demonstrate the wide range of biological activity that EW possesses, including improving cellular activity (viability, proliferation, migration, differentiation), inducing angiogenesis and tissue regeneration, and reducing inflammation [18,21].

Due to the relatively higher lipid concentration in the EY compared to other egg components, the yolk exhibits some different biological activities. The most notable properties are its high anti-inflammatory, anti-neurodegenerative, and anti-atherosclerotic properties. Uniquely, the EY’s plasma phase resembles human blood as it is rich in various peptides, antibodies (namely immunoglobulin Y (IgY)), and nutrients [34,41]. Phosvitin, a key protein of the insoluble phase of EY, has a wide variety of biological activities. It has been reported to be capable of inducing osteoblast differentiation and mineralization, metal chelating, antioxidant properties, and antimicrobial properties. Together, the various EY molecules have demonstrated a wide range of other biological activities, including enhancing wound healing, analgesic effects, neuroprotective and cardioprotective effects, and immune modulation [21,33,34,42,43].

## 2. Advanced Biomedical Applications of Egg Materials

### 2.1. Egg White (EW)

Unlike other protein-based biomaterials, EW is usable in its raw form for different applications thanks to its antibacterial properties [44]. EW has inherent antibacterial properties due to the presence of lysozyme, which can disrupt β-1,4 linkages in the bacteria barrier of both Gram-positive and Gram-negative bacteria [45]. Impressively, the EW’s antibacterial properties do not disappear in denatured forms; heat-denature at certain pH values and chemical-denatured treatments could even increase the hen EW lysozyme antibacterial activity against Gram-negative bacteria [46,47].

Aware of the excellent biocompatibility and antibacterial characteristics, researchers have utilized EW as a biomaterial in various applications, such as electro-spun fibers [44,48] and hydrogels [45,49,50,51] for wound healing, nanocomposites [52,53], and microfibers [47,54,55] for bone tissue engineering. As bacterial infections represent a major cause of the failure of medical implants and devices, coating material surfaces with lysozyme from EW could be considered a promising strategy [56].

EW can be collected from fresh eggs purchased from stores [23,57,58,59] and farms [60], or it can be purchased in a processed form such as egg powder from various companies [47]. There are different ways to process EW into a hydrogel; one method is altering its physicochemical properties such as changing the pH or temperature [45,50,61]. Another method is to introduce crosslinkers into EW, like glutaraldehyde (GA) and 1,4-butanediol diglycidyl ether (BDE) [16,62]. A third method, which is the most popular, is mixing and crosslinking EW with other molecules such as alginate, gelatin, and chitosan to form a composite or hybrid gel (Table 1) [10,49,57].

A water-insoluble EW hydrogel membrane was developed by Dong et al., which has applicable mechanical properties, softness, and elasticity [63]. The fabrication included a unique technology, unidirectional dehydration, where a film with nanopores is applied to allow the directional movement of water molecules from EW because of gravity [63,64]. The condensed EW proteins were then transformed into an organized structure and formed an EW hydrogel membrane with a heat treatment.

Without toxic chemicals involved, Ovomucin (OVN) could be isolated from raw EW using isoelectric precipitation method by altering the pH and ion strength [65,66]. OVN could be potentially used for creating desired pore sizes and increasing porosity in tissue engineering, given its excellent foaming property [67].

Luo et al. compared ovalbumin hydrogels crosslinked by three different crosslinkers: glutaraldehyde (GA), 1-ethyl-3-(3-dimethylaminopropyl) carbodiimide (EDAC), and 1,4-butanediol diglycidyl ether (BDE). As revealed by scanning electron microscope (SEM) images, various pore sizes and porosities were found in ovalbumin hydrogels with these crosslinkers. The BDE-crosslinked scaffold had the most uniform pore size distribution and a relatively narrow pore size range (50–160 mm). Only small pores could be seen on the surface of the GA-crosslinked scaffold, but its cross-sectional area pore size was found to be relatively uniform (20–180 mm). The EDAC-crosslinked scaffold had the most porous structure and the largest pore sizes (40–300 mm). Controllable pore sizes and porosity allow ovalbumin hydrogels to meet more needs of biomaterials and could potentially be applied to a larger range of biomedical applications.

Apart from synthetic materials, natural polymers have also been utilized to modify the mechanical properties of ovalbumin hydrogel. For example, flavonoids are able to promote the gelation of ovalbumin solutions, improving its strength and elasticity [68]. Another research group added gelatin to stabilize the OVN hydrogel for high porosity and implantability [65]. With an increase in OVN concentration, cell functions were improved, and more organized cytoskeletons were found in cells cultured on the scaffolds. In animal tests, a limited inflammatory response was found after implantation, indicating that it is a desirable candidate for various biomedical applications [65].

Zhao et al. generated hydroxypropyl chitosan–EW hydrogel (HPCS–EWH) with excellent biocompatibility and antibacterial properties. Moreover, as a hydrogel pad, it has desirable self-healing efficiency and mechanical strength, preventing deformation, damage, and cracking during its service life [49]. Furthermore, the HPCS–EWH provided a distinguished effect in promoting the healing of burn wounds regarding re-epithelialization, formation of granulation tissue, and tissue remodeling.

Chang and their colleagues developed a hydrogel with EW that has excellent stretchability, direct-writing 3D printability, and self-healing, without losing inherent bioactive elements [23,45]. Their hydrogel methodology combined two ways for EW gelation: alkalization and ionic crosslinking. In alkalization, carboxyl groups (-COOH) in EW proteins are deprotonated to -COO, and electrostatic repulsion becomes dominant, causing the breakdown of the original balance, followed by the reorganization of the protein chains and the formation of a solid hydrogel [69]. Later, they soaked the hydrogel in a culture medium with multivalent ions, such as Na^+^ and Ca^2+^, to establish a secondary network of ionic bonds. With ion crosslinking, the Young’s modulus of the hydrogel increased by 2.5-fold, and the pore size shrunk dramatically [45].

Unlike most bio-inks used for 3D printing that require gelatinization as soon as possible after printing to retain their shape and avoid fusion caused by gravity, this EW hydrogel could be directly written as 3D architecture [23]. Adding sodium hydroxide (NaOH) induces protein denaturation within the EW bio-ink, forming new bonds such as hydrogen bonding and hydrophobic interactions between protein chains. These dynamic bonds contribute to the shear thinning property, which is essential for successful direct ink writing. In direct ink writing, a bio-ink with the shear thinning property experiences stress surpassing its yield threshold when extruding through the nozzle. This mechanical strain induces a viscosity alteration, facilitating swift solidification subsequent to deposition [70]. Moreover, it was found that with different concentration ratios of EW and NaOH, the hydrogel’s mechanical properties can be easily tuned, such as the elongation ratio, tensile strength, and Young’s Modulus, making it a promising scaffold for use in various tissue engineering applications with different mechanical needs [23]. Notably, the same group further developed a 3D-printed sensor composed of EW hydrogel embedded with carbon nanotubes, which could record the radial augmentation index and the stiffness index of the cardiovascular system [23]. They also demonstrated that this hydrogel was capable of maintaining inherent bioactive elements after alkalization, supporting multiple biological activities in would healing, including angiogenesis and the promotion of collagen synthesis and assembly [45].

Beyond these findings, the integration of egg white into vascular scaffolds amplified cellular affinity. For example, Mousseau et al. utilized a thermally crosslinked EW matrix to successfully fabricate a pre-vascular network with endothelial and smooth muscle cells [71]. In the study by Wang et al., EW hydrogels were capable of enhancing blood vessel distribution, epidermal tissue formation, and increased presence of vascular endothelial growth factor (VEGF) in mouse wound healing [50]. Hu et al. developed a triple-layered vascular scaffold whose inner and outer layers were composed of a polycaprolactone-collagen composite, whereas the middle layer contained an EW and sodium alginate composite (EW-SA) [72]. Intriguingly, they fabricated the middle EW-SA layer by using a coaxial extruded-based printing technique. This technique leveraged a coaxial nozzle to dispense a calcium chloride (CaCl_2_) aqueous solution, functioning as the cross-linking agent for the alginate component of the hydrogel, thus achieving rapid layer formation.

EW has also garnered interest in bone regeneration research; its biocompatibility and antibacterial characteristics significantly improve the performance of bone/dental biomaterials, especially in in vivo studies [52,54,73,74,75]. Notably, Patty et al. developed a nanocomposite with EW, where ovalbumin significantly affected the pore sizes and porosity of their bone biomaterials, and controllable pore sizes and porosity could be achieved by involving different concentrations of EW [73]. Interestingly, another research group found that the presence of EW stimulated the expression of osteogenic genes, coding for bone sialoprotein and osteocalcin at mRNA level, indicating EW as a promotor of osteogenesis [75].

With the addition of alginate, EW has been employed as a biomaterial for salivary gland cell culture [10,57]. EW proteins in the hydrogel are locked by a network of alginate polymers when cross-linked with CaCl_2_ [76]. As the backbone of the material, the concentration of alginate affects various physical properties including stiffness, porosity, and pore size [77,78]. Ultimately, changes in these properties could influence the gene expression and cell behavior of embedded cells. We found that EW mixed with 2% *w*/*v* alginate (EW:Alginate (2%) = 2:1) showed the greatest cell viability, and the largest spheroids were found in 3% EWA (EW:Alginate (3%) = 2:1) [57]. The study showed that cells grown on EW-Alginate (EWA) scaffold had comparable spheroid size to cells grown on Matrigel^®^ over 3 weeks. Similarly, Duan et al. developed an EW dual crosslinked hydrogel with sodium hydroxide and CaCl_2_, and they found that increasing the concentration of calcium ions (from 0% to 4%) decreases the porosities of their hydrogels and the equilibrium swelling ratio [74].

In summary, researchers have crafted wound healing hydrogels, tissue-engineering scaffolds, and even 3D-printable constructs using egg white. Its antibacterial and bioactive attributes persist, holding potential for vascular scaffolds, bone regeneration, and salivary gland cell culture advancements. Its biological, mechanical, and physiochemical properties could be easily tuned by choosing different crosslinkers and partner molecules and adjusting their molecule concentrations. These easily accessible and cost-effective EW biomaterials hold great promise for advancing the field of tissue engineering and regenerative medicine, bridging the gap between scientific innovation and practical application.

**Table 1:** Different gelation methods of EW and the biomedical applications.

	Crosslinker/Gelation Methods	Crosslinks	Feature(s)	Application(s)	Reference(s)
Electrospun egg white/polyvinyl alcohol fiber	1-(3-dimethylaminopropyl)-3-ethyl carbodiimide (EDC)	Amide bonds between amines and carboxylic acid groups	Good spinnability (with 40–60% EW), mechanical strength, resistance to degradation, and good biocompatibility.	Wound healing	[48]
Ovalbumin-flavonoids self-assembled hydrogels	Flavonoids	(1) Hydrogen bond (between ovalbumin and puerarin/naringin/naringenin/genistein) (2) Hydrophobic interaction (between ovalbumin and daidzein)	Promoted the gelation of ovalbumin in low concentration; generated a hydrogel more elastic than viscous.	N/A	[68]
Ovalbumin hydrogel	1,4-butanediol diglycidyl ether (BDE)	Amide bonds	Uniform pore size	N/A	[62]
EW-alginate hydrogel	Calcium chloride	Ionic bonds	Easily reversible from hydrogel to solution	(1) 2.5D culture of human salivary acinar cells (2) 3D printing	[10,57,72]
EW-hydroxypropyl chitosan hydrogel	Chitosan	Intramolecular hydrogen bonding	Biocompatibility, Anti-inflammation Antibacterial property Self-healing	(1) 3D printing (2) To heal deep partial-thickness skin burn wounds	[49]
EW-gelatin hydrogel	Glutaraldehyde	Disulfide bonds	Tunable viscosity	(1) 2D hydrocolloid films (2) Microfluidic (3) 3D culture of Primary normal human dermal fibroblasts	[79]
EW hydrogel	Alkalization and ionic crosslinking	Hydrogen bonding and hydrophobic interactions, Ionic bonding.	Tunable mechanical properties (elongation ratio, tensile strength and Young’s Modulus)	(1) Direct-writing 3D printing (2) Wound healing (3) Electronic sensors	[23,45,50]

### 2.2. Egg Yolk (EY)

EY plasma is promising to be used as a biomaterial in tissue engineering, as it shares similarities with the plasma of human blood as both are enriched with peptides, antibodies, and nutrients. EY plasma can be obtained by centrifuging fresh EY at 15,400 RCF for 6 h [80]. However, the typical pH level of EY, which is around 6, may impede or have a negative impact on cell survivability. Our lab found that with the supplement of NaOH, commercial culture medium, PBS, or even EW, the EY’s pH could be altered from 6 to 7.3, which roughly resembles physiological pH, allowing for cells to survive in the presence of EY [81]. The gelation of EY plasma could be achieved by freezing the EY at −20 °C and then thawing it to 37 °C. It can stay in gel form at 37 °C, which allows the EY plasma to be 3D-printed as a tissue engineering scaffold with controllable geometries [80]. Specifically, EY plasma was used as a bio-ink in conjunction with EW and demonstrated cell proliferation of NS-SV-AC (a human salivary acinar cell line) without the support of a commercial culture medium over 14 days [81].

Similarly, the EY hydrogel produced by freeze-drying and crosslinked by transglutaminase exhibited excellent rheological behavior and bonding strength for wound dressing [82].

The biomedical applications of EY extend beyond its use as a hydrogel. Its fractions can also be isolated and incorporated into other materials. For instance, one study added EY oil to a chitosan gel to enhance the topical treatment properties for dermal burns, where its healing effects were statistically significant compared to Silverdin, a commercial product [83]. Moreover, as low-cost and biocompatible materials, EY and EW have been introduced to present technology to provide porous structures in microchannels of microfluidic devices, potentially applicable for biomedical diagnostics and environmental monitoring [84]. They could serve as perm-selective nano-porous materials to replace currently used nanostructures requiring sophisticated and expensive nanofabrication methods.

However, the most well-known biomedical application of EY is the utilization of its existing antibodies. Immunoglobulin Y (IgY), the primary immunoglobulin present in avian blood, can be transmitted to their offspring and accumulate in EYs, providing access to large amounts of antibodies non-invasively [85]. Therefore, EY is a well-known and reputable source for customized antibodies in health research and has been developed and used in commercial therapeutics and as diagnostic tools [86,87].

### 2.3. Eggshell (ES) and Eggshell Membrane (ESM)

As eggshell is rich in CaCO_3_ and easily obtained, it is considered an excellent source of calcium to generate hydroxyapatite (HA), the primary component found in bones. Studies have promoted HA synthesis by mixing calcium from ES with fibroin and alginate, fabricating an injectable hydrogel as a scaffold for bone regeneration [88]. Another group utilized EW and ES to create a novel biomimetic hybrid hydrogel, incorporating eggshell particles to enhance the hydrogel’s mechanical properties [89]. Moreover, the hydrogel was observed to promote osteogenic differentiation in vivo, as evidenced by the detection of essential biomarkers like Runx2 and Col-1.

ESM is also favorable in bone engineering research due to its collagen-based matrix and viscoelastic mechanical characteristics, offering a potential substitute for the extracellular bone structure. It has been characterized as porous and hydrophilic, and its biocompatibility can be improved by modifying its surface properties [90]. A novel 2D biomaterial for bone engineering, mineralized ESM, exhibited in vitro osteogenic differentiation and osteo-inductive properties [91]. The ESM was mineralized only on the outer surface, while the inner surface remained unmineralized. The mineral phase displayed chemical and structural characteristics resembling the inorganic phase of mineralized tissues, encouraging in vivo implantation studies and its potential use in dental/bone regeneration.

ESM is also considered an excellent material for wound dressing as it promotes higher secretion of VEGF and matrix metalloproteinase (MMP), enhancing the proliferation of keratinocytes, fibroblasts, and umbilical vein endothelial cells [92,93,94].

Interestingly, studies have suggested that ESM exhibits anti-aging properties by upregulating the decorin, MMP2, and type III collagen gene expressions, thereby enhancing the ECM environment within the papillary dermis of mice. Furthermore, a correlation was observed between elevated type III collagen levels and enhanced skin elasticity [95].

In summary, both ES and ESM hold biomedical potential for bone engineering and wound dressing. Abundant in CaCO_3_, ES supports osteogenic differentiation and hydroxyapatite synthesis. ESM, featuring collagen matrix and viscoelasticity, could be further applied in bone regeneration and skin health.

## 3. Conclusions and Future Remarks

This review highlights the structure, physiochemical properties, composition, biological activity, and application of the four major components of the chicken egg: the eggshell, eggshell membrane, egg white, and egg yolk. Compared to other biomaterial available on the market, the chicken egg is widely available, accessible, and affordable, making it an excellent choice for research. Furthermore, as discussed in this review, there are clear benefits of using the chicken egg in biomedical and TERM research due to its diverse biological activities. The use of chicken egg components in conjunction with other biomaterials allows researchers to create composite biomaterials with unique and diverse properties as needed for various studies [96].

However, researchers should be aware of the limitations of using the chicken egg as a biomaterial. The biggest limitation is the variation in the composition and physiochemical properties of the egg [97]. From batch to batch, eggs will exhibit slight variations in macronutrient and micronutrient properties. If using whole egg or whole egg components (e.g., ES, ESM, EW, or EY) for studies, the study outcomes may be affected by the changes of composition. This limitation may not apply if certain specific molecules are extracted for study purposes. Furthermore, depending on the chicken strain, diet, age, and temperature of storage, the chicken egg composition and quality of components may vary due to changes in pH and percentage of CO_2_ and water, thus ultimately affecting the quality and efficacy of select molecules and their function [98,99]. Researchers should seek to standardize—to the best of their ability—the source, storage condition, and timing of chicken eggs to optimize study accuracy and precision. Another limitation to consider is the allergen molecule, ovomucoid, found in the EW component. The egg is one of the most common food allergies; thus, as egg-based research progresses towards clinical studies, egg allergies should be a consideration.

There are multiple ways to crosslink egg proteins. Some common methods include using chemical crosslinkers such as glutaraldehyde [79] or NaOH [23,45], thermal crosslinking through temperature changes [80,81], and enzymatic crosslinking using enzymes like transglutaminase [100]. These methods create bonds between protein molecules, resulting in a more stable structure for various applications in biomaterials and tissue engineering. However, many crosslinking methods (such as alkalization and heating) and a variety of crosslinking agents (such as EDC or glutaraldehyde) also lead to protein denaturation. Therefore, future research and considerations are needed regarding the decreased efficacy of denatured egg proteins when crosslinked, as there is potential for active egg proteins to demonstrate improved or distinct performance in certain applications.

Our review has highlighted the wide usage of chicken egg research with promising and notable results. Continued research will further uncover the potential of chicken egg use in tissue engineering and regenerative medicine, whether it be use as a scaffold for cell growth and/or wound healing, as a vehicle for drug delivery, or as a source of nutrients. Future direction in egg research should look towards refining egg-based products and preparing for clinical studies and applications.
